# Genotyping versus phenotyping of non-ABO erythrocyte antigens in patients with the Mediterranean hemopathic syndromes: Effect of transfusion therapy

**DOI:** 10.1371/journal.pone.0251576

**Published:** 2021-07-06

**Authors:** Eman NasrEldin, Safaa A. A. Khaled, Nada O. Abdelhameed, Maha Atwa, Marwa M. Thabet, Khalid I. Elsayh, Sahar A. Elgammal

**Affiliations:** 1 Department of Clinical Pathology, Faculty of Medicine, Assiut University, Assiut, Egypt; 2 Department of Internal Medicine-Clinical Hematology Unit, Assiut University Hospital /Unit of Bone Marrow Transplantation, South Egypt Cancer Institute, Faculty of Medicine, Assiut University, Assiut, Egypt; 3 Department of Pediatrics, Faculty of Medicine, Assiut University, Assiut, Egypt; The Ohio State University, UNITED STATES

## Abstract

The Mediterranean hemopathic syndromes (MHS) are the most prevalent hemoglobinopathies in the Mediterranean basin. Transfusion therapy is the main therapy for these disorders, particularly for severe forms of the disease. Currently, pre-transfusion serological typing of erythrocyte antigens is the standard tool for reducing complications of transfusion in those patients. This study compared genotyping with phenotyping of non-ABO erythrocyte antigens in patients with MHS and assessed the effect of transfusion therapy on their results. One-hundred ninety-eight MHS patients were recruited, screened, and proven negative for allo-antibodies. They were grouped into two groups: (1) 20 newly diagnosed patients with no transfusion history and (2) 178 previously diagnosed patients undergoing transfusion therapy. Patients were interviewed and clinically examined. Full blood count (FBC) and high performance liquid chromatography (HPLC) were done for group 1 only. Genotyping and phenotyping of non-ABO erythrocyte antigens were performed for group 1, and 25 patients out of group 2 were propensity score-matched (PSM) with group 1. Both groups were gender and age matched; 55% and 74% of groups 1 and 2 had major disease, respectively. Insignificant differences were observed between genotyping and phenotyping of non-ABO erythrocyte antigens in group 1, while significant discrepancies and mixed field results were noted in group 2 patients. Discrepancies were obvious with JK^a^, JK^b^, and little c antigens. Conclusively, molecular typing is a powerful tool for pre-transfusion testing in chronically transfused MHS patients. This testing reduces incidence of transfusion reactions. JK^a^, JK^b^ and little c antigens are the most clinically significant non-ABO erythrocyte antigens.

## Introduction

The term “Mediterranean hemopathic syndromes” was suggested by Chini and Valeri to describe β-thalassemia, a group of hemoglobinopathies. It was found the most suitable as it describes the geographic distribution of the disorder and denotes the wide varieties of clinical presentations belonged to a single blood disorder [1, 2].

Recently the life expectancy of MHS patients prolonged, this was in part due to the availability of safe regular erythrocyte (RBC) transfusions and effective iron chelators. The best transfusion regimen that was proved effective in preventing organ damage, delayed growth, and bony changes, aimed to reach a hemoglobin (Hb) level of 9–10 and 13–14 g/dl as a pre- and post-transfusion levels, respectively [3]. Moreover, the recent introduction of Hydroxycarbamide as a therapeutic option for patients with MHS reduces the need for transfusion therapy in those patients [4]. However, regular blood transfusions are associated with many complications other than iron overload and infectious complications. Development of allo-antibodies is one of these serious complications, hence, pre-transfusion matching for non-ABO erythrocyte antigens is recommended in transfusion dependent patients [5, 6].

The International Society of Blood Transfusion and the Working Party committee on terminology for erythrocyte antigens had identified 342- erythrocyte antigen and 35 blood group systems. The most prevalent allo-antibodies were found directed towards the following blood group systems, RH (D, C, E, c and e), KELL (K), Duffy (FY) (Fya and Fyb), Kidd (JK) (Jka and Jkb) and the MNS (M, S and s) [6].

The conventional procedure used to identify non-ABO erythrocyte antigens is by serological phenotyping that relies on the interaction of a polyclonal or monoclonal antibodies with the erythrocyte surface antigen [7]. Advancement in genomics allowed the use of deoxyribonucleic acid (DNA)-based molecular techniques in transfusion medicine. In 2000s the EU Blood Gen group was recognized aiming to improve transfusion compatibility and patient safety through standardization and validation of molecular genotyping techniques. The polymerase chain reaction (PCR) is a vital tool for erythrocyte genotyping, moreover PCR and gel electrophoresis are easy, low cost, and flexible methods for amplifying specific target DNA. Allele Specific PCR (AS-PCR), Multiplex-PCR, and Restriction Fragment Length Polymorphism-PCR (RFLP-PCR) techniques are commonly used to identify erythrocyte polymorphisms [8–10].

This study aimed to compare genotyping with phenotyping techniques for detection of non-ABO erythrocyte antigens in patients with the MHS, and assess the effect of transfusion therapy on their results. The main study target was to improve blood matching techniques and reduce complications of transfusion therapy in those patients, particularly development of allo-antibodies.

## Patients and methods

The study sample size was calculated using the online calculator tool (www. Calculator.net) and considering prevalence of MHS in Egypt 1.3–2% [11], 95% confidence interval and margin of error 5. The minimum required sample was 20- patients and the maximum was 31. However as this was a case control study all new patients and old patients who attended at our institutions over one year were recruited. This was to compensate for dropout, refusals and case control mismatch.

### Study settings and patients

This study included 198-patient with the MHS who were attending the Thalassemia Center at Assiut University Children’s Hospital, the Clinical hematology Unit at Internal Medicine department, Assiut University Hospital (AUH), and the Hemolytic anemia laboratory at the Clinical Pathology Department, AUH, Assiut, Egypt. Patients were cross sectionally recruited in the period 1st of Jan. to 31st of Dec. 2019. They were grouped into, group 1: 20-newly diagnosed patients who did not receive blood transfusion before (act as controls), and group 2: 178 –patients who came for regular blood transfusions. 25-patient out of group 2 undergone propensity score matching (PSM) of age and gender with group 1, for comparison. All patients were of the same ethnic group, Egyptians living at Upper Egypt.

**Patients’** **inclusion criteria:**
  - Willingness to participate in the study
  - Patients with MHS of major or intermediate type
  - Regular transfusions for ≥ 1-year (for group 2 only)
  - Absence of allo-antibodies
**Patients’** **exclusion criteria:**
  - Patients with minor form of the disease
  - MHS combined with other types of qualitative hemoglobinopathies e.g. S hemoglobin.
  - Presence of chronic infection or inflammation.

### Procedures and methods

#### Medical history and clinical examination

All patients were interviewed and clinically examined to assess eligibility for the study and collect data. The collected data included patients’ demographic characteristics as their age, gender, residence and consanguineous marriage of their parents. Disease history was obtained to address transfusion dependency. Splenic examination was done and its size was measured from below the left costal margin. It was considered huge if its tip reaches the umbilicus or measures ≥ 20 cm below the costal margin. Based on patients’ clinical presentation the MHS were categorized as major disease if they have severe form of the disease (transfusion dependence, splenomegaly, extramedullary hematopoiesis, bony changes, profound hemolysis) and intermediate disease in those with moderate disease severity (transfusion independence, splenomegaly, hemolytic anemia patient).

#### Full blood count (FBC) and high performance liquid chromatography (HPLC), for group 1 only

Blood samples were withdrawn for FBC and HPLC for group 1 only. FBC was performed on CELL-DYN 3700 (Abbott-Germany) and blood film was stained by leishman staining to assess morphology of red blood cells. Reticulocyte count was detected by Brilliant Cresyl Blue stain.

**Fig 1:**
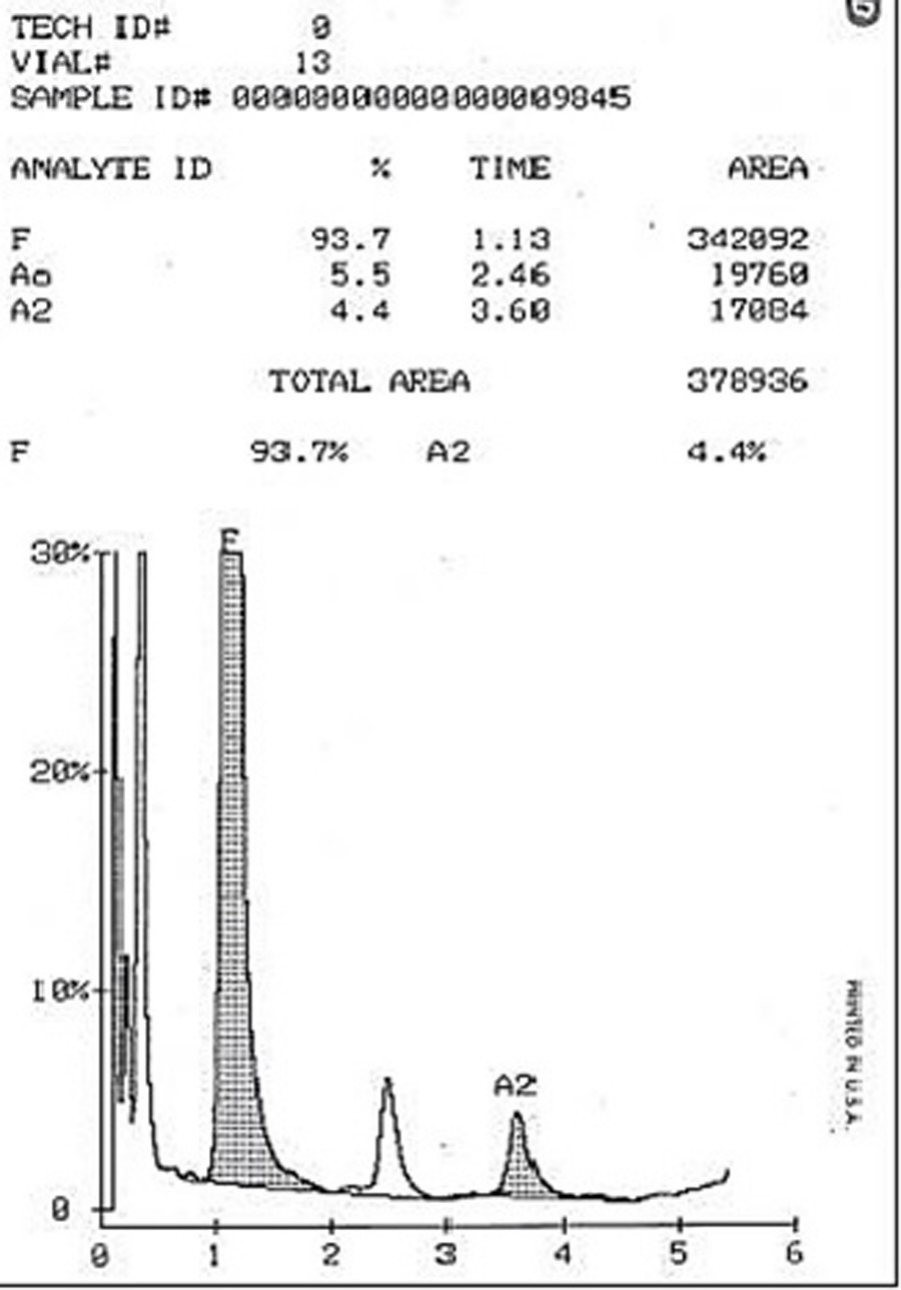
High Performance Liquid Chromatography (HPLC) report of a case with major disease.

The D10 automated hemoglobin testing system was used for chromatographic separation of the analysts by ion exchange HPLC (BIO-RAD laboratories Inc., Hercules, CA, USA). Venous samples were automatically diluted on the D-10 then injected into the analytical cartridge. Hemoglobins were separated according to their ionic interactions with the cartridge material. The separated hemoglobins then passed through the flow cell of the filter photometer, where changes in the absorbance at 415 nm were measured. Fig 1 showed HPLC report of a case with major disease.

#### Serological phenotyping of non- ABO erythrocyte antigens in the study patients

*Sampling*. 3-ml of venous blood were collected in two tubes each containing tripotassium EDTA anticoagulant. One was used for phenotyping and the other tube was frozen then stored to be used later for extraction of DNA.

Kp^a^, Kp^b^, Jk^a^, Jk^b^, M, N, antigens were assessed together, also Fya, Fyb, S, s constituted a group and the last group was D, C, c, E, e, kell.

*Procedure for Kp*^*a*^, *Kp*^*b*^, *Jk*^*a*^, *Jk*^*b*^, *M*, *N*. Using ID-card (Anti-Kpa, Anti-Kp^b^, Anti-Jk^a^, Anti-Jk^b^, Anti- M, Anti-N) with 6 microtubes containing polyclonal anti-Kpa, anti-Kp^b^, anti-Jk^a^, anti-Jk^b^, anti- M, anti-N antibody respectively from human serum within the gel matrix respectively (Diamed, Switzerland).

Preparation of the blood sample:

5% red suspension was prepared in ID-Diluent 1 as follows:

1. 0.5 ml of Diluent 1was dispensed into a clean tube.
2. 50 μl of whole blood was added and mixed gently.
3. The red suspension was incubated for 10 minutes at room temperature.

Test procedure:

1. The microtubes of the ID-card “DiaClon Anti-Kp^a^, Kp^b^,……. etc.” were identified with patient’s name
2. The aluminum foil was removed from microtubes as required
3. 10 or 12.5 μl of the red suspension was dispensed to the microtube
4. The ID-card was centrifuged for 10 minutes in the ID-centrifuge

*Procedure for Fya*, *Fyb*, *S*, *s*. Using ID-card (Anti-Fya, Anti-Fyb^b^, Anti-S, Anti-s) with 6 microtubes containing polyspecific anti-human globulin serum (rabbit anti-IgG and monoclonal anti-C3d, cell line C139-9) within the gel matrix (Diamed, Switzerland).

Preparation of the blood sample:

0.8% red suspension was prepared in ID-Diluent 2 as follows:

1. 1 ml of Diluent 2 was dispensed into a clean tube.
2. 20 μl of whole blood was added and mixed gently

Test procedure:

1. The microtubes of the ID-card “DiaClon Anti-Fya, Fyb,……. etc.” were identified with patient’s name
2. The aluminum foil was removed from microtubes as required
3. 50 μl of the red suspension was dispensed to the microtube
4. 50 μl of the corresponding ID-test serum was added.
5. The ID-card was incubated at 37°c for 15 minutes.
6. The ID-card was centrifuged for 10 minutes in the ID-centrifuge.

*Procedure for*, *C*, *c*, *E*, *e*, *kell*. Using ID-card “Diaclon Rh-subgroups+K” contains monoclonal antibodies anti-C (cell line MS-24), anti-c (cell line MS-33), anti-E (cell line MS-260), anti-e (cell lines MS-16, MS-21, MS-63) and anti-K (cell line MS-56) within each gel matrix. The microtube (ctl) is the negative control (Diamed, Switzerland).

Preparation of the blood sample:

5% red suspension was prepared in ID-Diluent 2 as follows:

1. 0.5 ml of ID-Diluent 2 was dispensed into a clean tube.
2. 50 μl of whole blood or 25μl of packed cells was added and mixed gently

Test procedure:

1. The ID-card was identified with the patient’s name.
2. The aluminum foil was removed
3. 10 or 12.5 μl of the patient’s red cell suspension was pipetted to the microtubes, C, c, E, e, kell, ctl
4. The ID-card was centrifuged for 10 minutes in the ID-centrifuge

**Fig 2:**
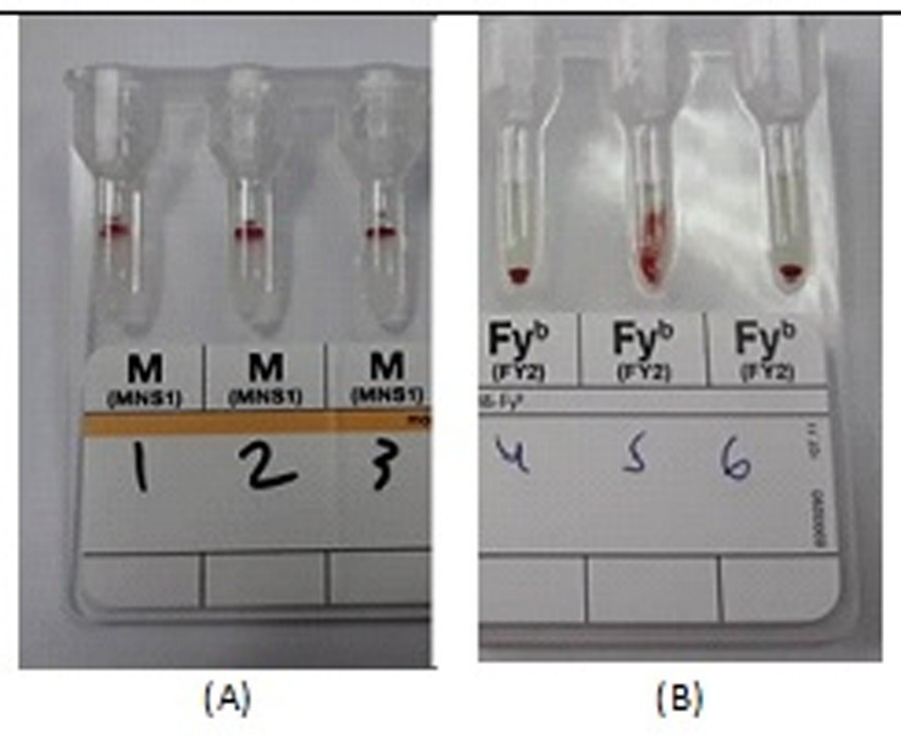
Phenotyping of Non-ABO erythrocyte antigens performed on ID-card “DiaClon Anti-M (A) revealed positive results in patients no. 1,2,3 and another one on ID-card “DiaClon Anti-Fy^b^” (B) revealed negative results in patients 4 &6 and positive in 5.

For all antigen groups we followed others and interpreted the results as positive when the agglutinated erythrocytes making a red line on the gel surface or the agglutination dispersed in the gel. Negative if there was a solid mass of erythrocytes on the bottom of the microtube. Mixed field was observed if there was a dual erythrocyte population appearance, as in Fig 2 [10, 12].

#### Genotyping of non- ABO erythrocyte antigens of the study patients

DNA was extracted from blood samples of both groups using Gene JET Whole Blood Genomic DNA Purification Mini Kits (catalog no. #K0781, #K0782 Thermo Scientific, USA) and following manufacturer’s instructions, as described in S1 File. Then genotyping for erythrocyte antigens was done by conventional qualitative PCR. Some were done in multiplex reaction mixes of up to six amplification targets per mix and the length of the product varied from 259–713 bp. Others were performed in single reaction, using primers sets supplied by Biosearch technologies-LGC, USA. The PCR reagents, conditions and cycling conditions were detailed in the S1 File. Products of PCR were analyzed by gel electrophoresis which was run for 1-h at 110-volts. An UV trans- illuminator was used for inspection of the gel and a DNA marker was used to assess the molecular size of the PCR product. Details of genotyping technique were provided in the S1 File.

### Ethical statement

The study was approved by the research ethical committee of the Faculty of Medicine at our institution, and was in accordance with the Declaration of Helsinki. All patients signed a written informed consent; consent was obtained from patients’ guardians for those younger than 18-years old.

### Statistical analyses

Data were collected then analyzed with SPSS v 21 (SPSS Inc., Chicago, Illinois, USA). Microsoft Excel and GraphPad Prism V5. software packages were also used for creation of figures. Quantitative and Qualitative variables were expressed as mean, median, range, and frequency, percentage; respectively. The Chi-square test was used to examine the relationship between qualitative variables and unpaired T-test to analyze differences of quantitative variables among the study groups. Significance was considered when P-value <0.05. Direct counting was used to estimate genotype and phenotype frequency and the percentage was calculated by dividing the number of patients.

## Results

### Characteristics of the study patients (group 1 = newly diagnosed MHS patients and group 2 = previously diagnosed MHS patients on regular transfusion therapy without allo-antibodies), Table 1

**Table 1:** Demographic and clinical characteristics of the study groups and their ABO and Rhesus phenotypes.

Variable	Group 2 (n = 178)	Group 1(n = 20)	P value
**Demographics**			
**Age**			
Mean ± SEM	11.87 ± 0.3702	15.38 ± 1.608	0.0048**^#^
Mean difference	-3.502 ± 1.227		
**Gender**			
Male (n (%))	126 (70.8)	10 (50%)	0.053
**Governorate (n (%)**			
Assiut	74(41.6)	9(45)	
Sohag	69(88.5)	5(25)	
Luxor	16(9)	3(15)	0.66
Red Sea	12(6.7)	2(10)	
Aswan	7 (3.9)	1(5)	
**Parents’ Consanguinity (n (%))**	123(69.1)	14 (70)	0.58
**Clinical characteristics (n(%))**			
MHS major disease	127(71.3)	11(55)	0.11
Transfusion dependent	124 (69.6)	12(60)	0.26
Splenomegaly			
Huge	130(73)	10(50)	
Moderate	37(20.8)	8(40)	0.09
Mild	11(6.2)	2(10)	
**ABO group (n (%))**			
A	41(23)	8(40)	
B	70(39.3)	7(35)	0.39
AB	39(21.9)	3(15)	
O	28(15.7)	2(10)	
**RH positive (n (%))**	155(87.9)	19(95%)	0.30

*X*^*2*^- test,

^#^ Unpaired T-test,

** P <0.005. MHS: Mediterranean Hemopathic syndrome, SEM: standard error of the mean, RH: Rhesus factor.

A total of 198- MHS patients were enrolled in the study, 20-newly diagnosed (group 1) and 178- old cases (group 2). Analysis of the collected data showed significant differences in age and gender between the two groups, P = 0.004 and 0.053, respectively. However, insignificant differences were noted between the two groups as regard residency, consanguinity of their parents, MHS disease subtype, transfusion dependency, splenomegaly and ABO and Rhesus phenotypes. Accordingly group 2 patients were subjected to gender and age matching with group 1 using Propensity score matching. Only 25- of group 2 were found matched with group 1 and were used for further analyses.

**Fig 3:**
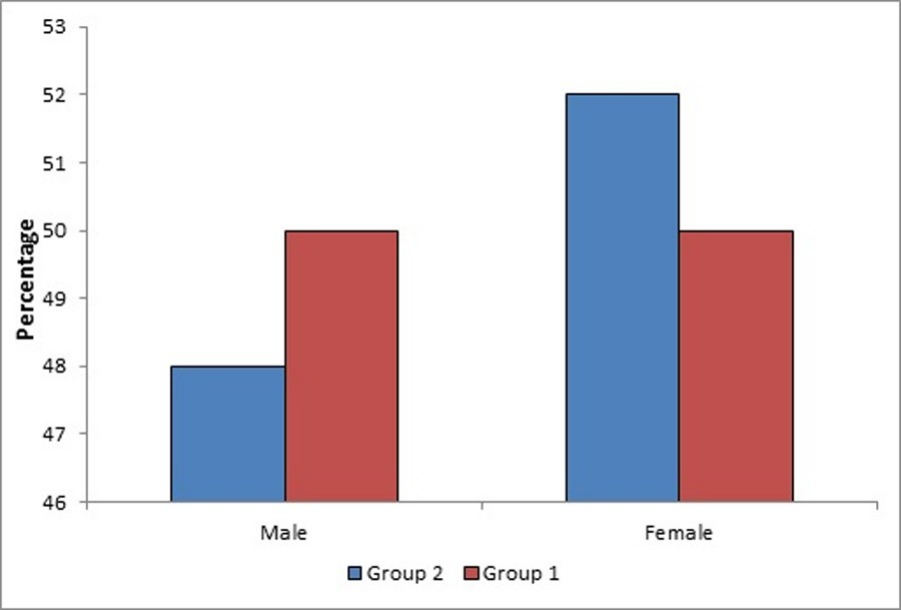
Gender distribution among the studied Mediterranean hemopathic syndromes patients’ groups.

The age range of Group 1 patients was from 1–29 years, and the male to female ratio was 1:1. 55% and 45% of them were with major and intermediate disease respectively. The median age of Group 2 patients who subjected to PSM with group 1 was 13, range from 2–19 years, and the male to female ratio was 1:1.08. 76% and 24% of them were with major and intermediate disease respectively. Both groups were matched as regard age and gender and significantly different as regard type of disease, P-values were 0.45, 0.11, and 0.03 respectively. Fig 3 illustrated gender distribution of the studied groups.

### Distribution of non-ABO erythrocyte antigens among the study patients

**Fig 4:**
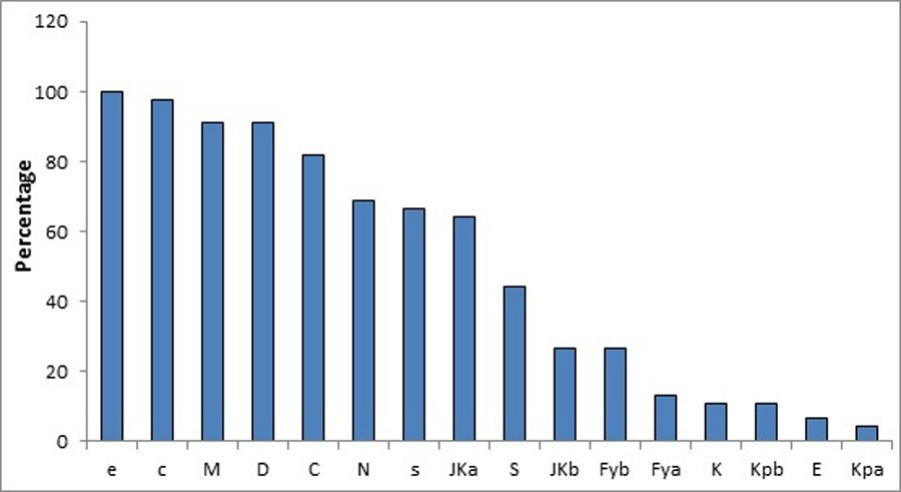
Distribution of 16 non-ABO erythrocyte antigens among the total study patients.

Fig 4 showed Distribution of non-ABO erythrocyte antigens among the studied groups. The most frequent antigens among both groups were e, c, M and D (100%, 97.7%, 91.1% and 91.1%) in order.

### Genotyping versus phenotyping of non-ABO erythrocyte antigens in the studied groups

Table 2 showed comparison between the results of genotyping and phenotyping of different non-ABO erythrocyte antigens in the study patients’ groups. In group 1 patients there were insignificant differences between results of genotyping and phenotyping of different blood group systems except for the Kell system. In this system the detection of Kp^b^ antigen by genotyping was significantly lower (5%) than phenotyping (20%); with P value 0.04.

**Table 2:** Comparison between the results of genotyping and phenotyping of the 16 non-ABO erythrocyte antigens in the study patients’ groups.

	Group 1(newly diagnosed MHS patients)			Group 2 (previously diagnosed MHS patients)		
Antigen	Phenotyping	Genotyping	*P* value	Phenotyping	Genotyping	*P* value
**RH system**						
**D**	17 (85%)	18 (90%)	0.55	21 (84%)	23 (92%)	0.45
**C**	17 (85%)	17 (85%)	---	19 (76%)	20 (80%)	0.56
**c**	20 (100%)	20 (100%)	---	**16 (64%)**	**24 (96%)**	**< 0.001**
**E**	0	1 (5%)	0.34	1 (4%)	2 (8%)	0.55
**e**	20 (100%)	20 (100%)	---	23 (92%)	25 (100%)	0.06
**Kidd system**						
**JK**^**a**^	11 (55%)	13 (65%)	0.23	**8 (32%)**	**16 (64%)**	**< 0.001**
**JK**^**b**^	5 (25%)	7 (35%)	0.14	**1 (4%)**	**5 (20%)**	**0.04**
**MNS system**						
**M**	18 (90%)	17 (85%)	0.08	**19 (76%)**	**24 (96%)**	**<0.001**
**N**	12 (60%)	15 (75%)	0.15	**10 (40%)**	**16 (64%)**	**< 0.001**
**S**	12 (60%)	11 (55%)	0.23	12 (48%)	9 (36%)	0.06
**s**	16 (80%)	14 (70%)	0.10	17 (68%)	16 (64%)	0.09
**Kell system**						
**K**	1 (5%)	2 (10%)	0.09	0	3 (12%)	0.06
**Kp**^**a**^	1 (5%)	1 (5%)	---	0	1 (4%)	0.33
**Kp**^**b**^	**4 (20%)**	**1 (5%)**	**0.04**	5 (20%)	4 (16%)	0.09
**Duffy system**						
**Fy**^**a**^	3 (15%)	2 (10%)	0.12	2 (8%)	4 (16%)	0.35
**Fy**^**b**^	3 (15%)	4 (20%)	0.06	5 (20%)	8 (32%)	0.07

Data was expressed as frequency (percentage). P- Value was considered significant if < 0.05.

On the contrary significant differences were noted between results of genotyping and phenotyping of 3-blood group systems in group 2 patients. The 3-systems were the MNS, Kidd and RH where the detection by genotyping of M, N, JKa, JKb, and c antigens was significantly higher than phenotyping (P value < 0.001, < 0.001, < 0.001, 0.04, and < 0.001; respectively).

### Genotyping versus serological phenotyping of non–ABO erythrocyte antigens in the study MHS patients, and the effect of transfusion therapy

This study analyzed the effect of transfusion therapy on the results of serological phenoty ping and genotyping for the 16 non-ABO blood group antigens involved in the study. By comparing the results of both methods among the studied groups; newly diagnosed patients without history of blood transfusion (group 1) and previously diagnosed patients on regular transfusion therapy (group 2).

**Fig 5:**
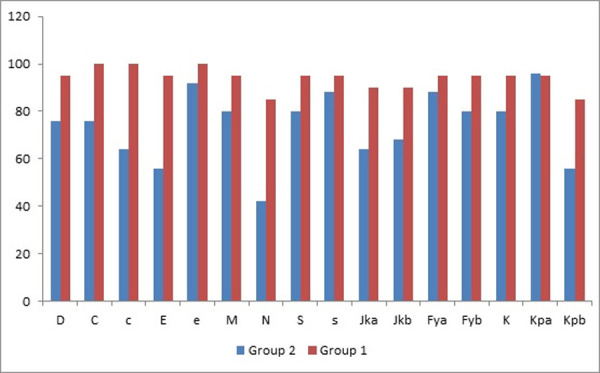
Frequency of identical results of phenotyping and genotyping of each studied non-ABO- erythrocyte antigen among the studied Mediterranean hemopathic syndromes patients’ groups.

Table 3 showed that the degree of identity between phenotyping and genotyping in all antigens of the Rh, MNS, Kidd, and Duffy systems together with K and Kp^b^ of the Kell system, were higher in the newly diagnosed group1 patients. Fig 5 revealed that the percent of identity between the results of phenotyping and genotyping was higher among group 1 than group 2 as regarding results of all studied blood group systems. Furthermore the highest frequency of identity of results between phenotyping and genotyping was reported for Duffy system (88.9%) while the least frequency was reported for Kidd system (76.7%).

**Table 3:** Analysis of the effect of transfusion therapy on the results of phenotyping and molecular genotyping of non-ABO erythrocyte antigens in the studied Mediterranean hemopathic syndromes patients’ groups.

Antigen	Previously diagnosed MHS patients with transfusion history (group 2)	Newly diagnosed MHS patients with no transfusion history (group 1)	*P*–value
**RH system**			
**D**			
Identical	19 (76%)	19 (95%)	
Discrepancy	6 (24%)	1 (5%)	**< 0.001**
Mixed field	0	0	
**C**			
Identical	19 (76%)	20 (100%)	
Discrepancy	1 (4%)	0	**< 0.001**
Mixed field	5 (20%)	0	
**C**			
Identical	16 (64%)	20 (100%)	
Discrepancy	2 (8%)	0	**< 0.001**
Mixed field	7 (28%)	0	
**E**			
Identical	14 (56%)	19 (95%)	
Discrepancy	0	1 (5%)	**< 0.001**
Mixed field	11 (44%)	0	
**E**			
Identical	23 (92%)	20 (100%)	
Discrepancy	2 (8%)	0	**< 0.001**
Mixed field	0	0	
**MNS system**			
**M**			
Identical	20 (80%)	19 (95%)	
Discrepancy	0	1 (5%)	0.05
Mixed field	5 (20%)	0	
**N**			
Identical	13 (42%)	17 (85%)	
Discrepancy	3 (12%)	3 (15%)	**< 0.001**
Mixed field	9 (36%)	0	
**S**			
Identical	20 (80%)	19 (95%)	
Discrepancy	5 (20%)	1 (5%)	0.05
Mixed field	0	0	
**S**			
Identical	22 (88%)	19 (95%)	
Discrepancy	1 (4%)	1 (5%)	0.22
Mixed field	2 (8%)	0	
**Kidd system**			
**Jk**^**a**^			
Identical	16 (64%)	18 (90%)	
Discrepancy	1 (4%)	2 (10%)	**< 0.001**
Mixed field	8 (32%)	0	
**Jk**^**b**^			
Identical	17 (68%)	18 (90%)	
Discrepancy	0	2 (10%)	**< 0.001**
Mixed field	8 (32%)	0	
**Duffy system**			
**Fy**^**a**^			
Identical	22 (88%)	19 (95%)	
Discrepancy	0	1 (5%)	0.56
Mixed field	3 (12%)	0	
**Fy**^**b**^			
Identical	20 (80%)	19 (95%)	
Discrepancy	2 (8%)	1 (5%)	0.05
Mixed field	3 (12%)	0	
**Kell system**			
**K**			
Identical	20 (80%)	19 (95%)	
Discrepancy	3 (12%)	1 (5%)	0.06
Mixed field	2 (8%)	0	
**Kp**^**a**^			
Identical	24 (96%)	19 (95%)	
Discrepancy	1 (4%)	1 (5%)	0.44
Mixed field	0	0	
**Kp**^**b**^			
Identical	14 (56%)	17 (85%)	
Discrepancy	1 (4%)	3 (15%)	**0.04**
Mixed field	10 (40%)	0	

Data was expressed as frequency (percentage). P -value was considered significant if < 0.05.

On the contrary, the degree of discrepancy in results for D, C, c, e, S, Fyb, and K antigens between the two methods were higher in group 2 than group 1 patients. While the percent of discrepancy in results for JKa, JKb, Fya, Kpa and Kpb, antigens between the two methods were higher in group 1 than group 2 patients, as in Table 3.

Table 4 showed that the percent of discrepancy in results of phenotyping and genotyping among the previously diagnosed group (group 2) was higher for all non-ABO blood group systems than that among the newly diagnosed group (group 1) except for Kidd and Duffy systems. It was clear that the highest frequency of discrepancy was reported for MNS system (8.3%) followed by Kell system (7.4%), while the least frequency was reported for Duffy system (4.4%).

**Table 4:** Frequency of discrepancy of results between phenotyping and genotyping among each non-ABO blood group system in the studied MHS groups.

Non-ABO System	Previously diagnosed patients (group 2)	Newly diagnosed patients (group 1)	Total
**Rh**	11/125 (8.8%)	2/100 (2%)	5.7%
**MNS**	9/100 (9%)	6/80 (7.5%)	8.3%
**Kidd**	1/50 (2%)	4/40 (10%)	5.5%
**Duffy**	2/50 (4%)	2/40 (5%)	4.4%
**Kell**	5/75 (6.6%)	5/60 (8.3%)	7.4%

Data was expressed as frequency (percentage).

**Fig 6:**
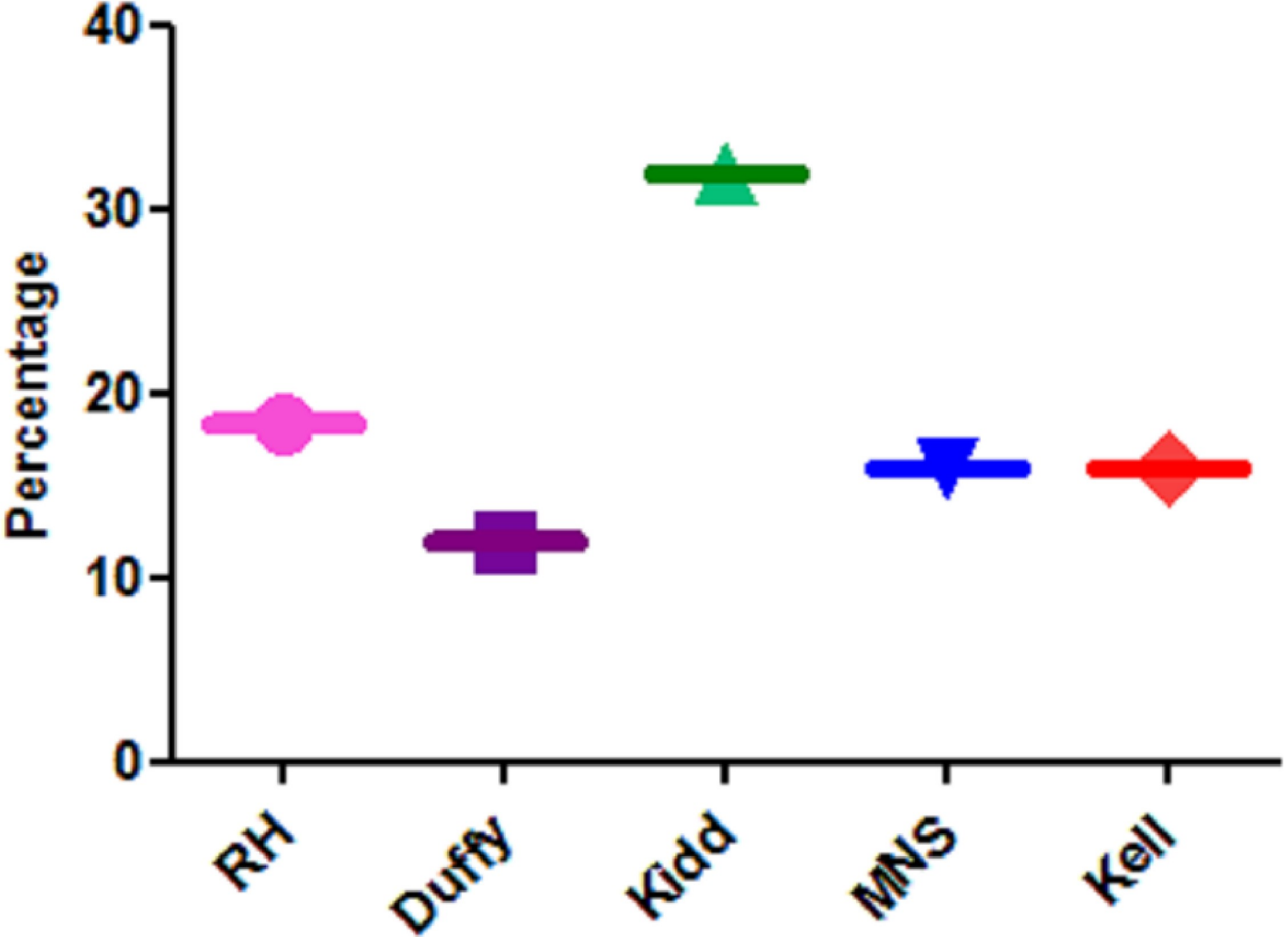
Frequency of mixed field results among group 2 patients for each non-ABO blood group system.

On the other hand mixed filed results were observed only in some group2 patients (previously diagnosed group on transfusion therapy) for C, c, E, M, N, s, Jk^a^, Jk^b^, Fy^a^, Fy^b^ and Kp^b^ antigens, as in Table 3. Fig 6 illustrated the frequency of mixed field results in group 2 patients for all non-ABO blood group systems. It showed a total percent 18.3 (73/400), and the highest frequency for Kidd system 32% (16/50) followed by Rh, MNS and Kell systems 18.4% (23/125), 16% (16/100) and16% (12/75) respectively, while the least frequency was for the Duffy system 12% (6/50).

## Discussion

Proper blood typing of donors and patients is crucial to prevent complications of transfusion therapy. There are various erythrocyte antigens other than the ABO-blood group system that are capable of causing transfusion problems [12, 13]. Although serological phenotyping is the standard tool for identification of blood group systems, results may be affected by presence of recently transfused erythrocytes, its coating with immunoglobulin, or any polyagglutination with mixed-field undetermined results [7].

This study compared genotyping and phenotyping techniques for non-ABO erythrocyte antigens in patients with the MHS, and assessed the effect of transfusion therapy on their results. As our target was to improve transfusion therapy the vast majority of our cohort was transfusion dependent with major and to a lesser extent intermediate disease.

In the current study, results of phenotyping of non-ABO erythrocyte antigens of the Duffy, Rh, Kell, MNS and Kidd blood group systems among the study patients, showed a distribution that was albeit consistent with a study in the same ethnic group and country, Egypt [14].

To obtain more accurate results we analyzed differences of genotyping and phenotyping procedures in patients’ groups separately (group 1: newly diagnosed patients without history of blood transfusions and group 2: previously diagnosed on transfusion therapy). Notably, no significant differences observed in the results of both methods except for Kpb antigen in group 1 patients. These results were similar to the results of other studies among blood donors and healthy volunteers [9, 15]. Our results together with others indicate no superiority of genotyping over phenotyping when used to identify blood group typing for subjects with no history of prior transfusion and negative screening antibody test. This means that genotyping is not recommended in those subjects particularly it is a more expensive, complex and lengthy procedure [16].

On the contrary in group 2 patients significant differences were noted between results of genotyping and phenotyping of some non-ABO erythrocyte antigens. These findings confirmed the findings of others [17, 18] and proved superiority of genotyping technique over phenotyping for RBCs antigen detection in regularly transfused MHS patients. Moreover, Belsito et al. and others reported that patients who receiving RBCs transfusion based on genotyping showed longer in vivo RBCs life span, higher post-transfusion Hb levels and reduced transfusion reactions [19, 20].

When we studied the effect of transfusion therapy on results of phenotyping and genotyping of 16 non-ABO erythrocyte antigens we found 6.7% discrepancy between phenotyping and genotyping among group 2 patients. The highest frequency of discrepancy was reported for MNS system, followed by Rh, Kell and the least frequency was for Kidd followed by Duffy system. This was nearly similar to another study in thalassemia (5.4%) and lower than in sickle cell disease (22%) [15, 20]. However, our results of the frequency of discrepancies of different blood group systems were inconsistent with other studies where Duffy system had the highest frequency of discrepancies between phenotyping and genotyping of erythrocyte antigens [8, 20]. These differences between other studies and our study are likely due to different sample size.

Our study showed that the percent of identity (even positive or negative results) between the two methods is higher in newly diagnosed than in previously diagnosed group for all blood group antigens. However, we reported mistyping of non-ABO erythrocyte antigens when haemagglutination was performed alone in patients who were on regular transfusion therapy.

In accordance with others mixed-field results were observed in our study in multiple antigens, in all non-ABO blood group systems, when typing was done with serological methods. It was reported only among group 2 patients with transfusion history. In which Kidd system has the highest frequency followed by Rh, MNS and Kell and the least frequency was reported for the Duffy system. This generates difficulties in accurate phenotyping of patient’s blood. Thus may upsurge the risk of alloimmunization as some of these blood group antigens can stimulate clinically significant antibodies and cause transfusion reactions [9, 21].

An important question is which is the most immunogenic non-ABO erythrocyte antigen? In this study only the following antigens little c, JK^a^ and JK^b^ showed obvious differences in their detection by serological phenotyping and genotyping and have a remarkable effect in transfusion. This was consistent with Tomey and Stack who considered Jk^a^ as the 4^th^ most immunogenic antigen after C^w^, K and Lu^a^ in multitransfused males [22]. Their detection in pre-transfusion serology is difficult, as they are weakly reactive. After transfusion of antigen+ve red cells, the antibody titer rises rapidly and hemolyzes the transfused red cells [23]. Moreover, Siddon et al. showed that JK antibodies, mainly anti-Jk^a^ are responsible for 29% of the delayed post- transfusion reactions in 2 hospital reviews [24].

Many researchers found that the little c is the most prevalent and clinically significant RH- antigen following D and cause delayed transfusion reactions and hemolytic disease of the new born [25–27].

The most important limitation of the current study was the small sample size. This was obvious although the study included 198- patients as most of the study procedures and analyses were applied to only 45- of the whole study sample. However, this sample size was reasonable and albeit suitable for the study as genotyping of erythrocyte antigens is a complex, lengthy and expensive procedure.

## Conclusion

This study analyzed the advantages and limitations of genotyping and phenotyping techniques for detection of non-ABO erythrocyte antigens in patients with the MHS and the effect of transfusion therapy on the results of both methods. Study results showed that pre-transfusion serology could be enough to avoid complications of transfusion in those with no history of previous transfusion. On the contrary genotyping is the perfect method for multitransfused patients. However we noted that both are crucial, complementary, and contributing positively to the identification of patient’s phenotype. Also, this study concluded that accurate Kidd system and little c antigen typing is highly significant in red cell transfusions.

## Supporting information

S1 File (DOCX)
