# Supplementary material for: Genotyping versus phenotyping of non-ABO erythrocyte antigens in patients with the Mediterranean hemopathic syndromes: Effect of transfusion therapy
Source: PLoS One. 2021 Jul 6;16(7):e0251576. doi: 10.1371/journal.pone.0251576 (PMC8260000; doi:10.1371/journal.pone.0251576)
Supplement: S1 File — (DOCX) [file pone.0251576.s001.docx]

**Supplementary material of article entitled: Genotyping versus phenotyping of non-ABO erythrocyte antigens in patients with the Mediterranean Hemopathic Syndromes: effect of transfusion therapy**

Eman NasrEldin ^1¶^, Safaa A A Khaled ^2¶^*, Nada O Abdelhameed^1¶^, Maha Atwa ^1¶^, Marwa M Thabet^1^ ^&^, Khalid I Elsayh ^3¶^, Sahar A Elgammal ^1¶^

^1^Department of Clinical Pathology, Faculty of Medicine, Assiut University , Assiut, Egypt.

^2^Department of Internal Medicine- Clinical Hematology Unit, Assiut University Hospital /Unit of Bone Marrow Transplantation, South Egypt Cancer Institute , Faculty of Medicine, Assiut University, Assiut, Egypt.

^3^Department of Pediatrics, Faculty of Medicine, Assiut University , Assiut, Egypt.

***** Corresponding author

Email: [safaakhaled2003@gmail.com](mailto:safaakhaled2003@gmail.com), safaakhaled1997@aun.edu.eg

**Methods:**

**a) Genotyping of Non-ABO erythrocyte antigens in the study groups**

**Extraction of DNA from whole blood samples**

**Procedure:**

- Step (1): 20 μl of proteinase K solution were added to 200 μl of whole blood, and mixed by vortexing then 400 μl of lysis solution was added and mixed thoroughly by vortexing to obtain a uniform suspension.
- Step (2): The sample was incubated at 56°C for 10 minutes while vortexing occasionally used.
- Step (3): 200 μl of ethanol (96-100%) were added and mixed by pipetting.
- Step (4): The prepared mixture was transferred to the spin column and centrifuged for 1 min at 8,000 rpm. The collection tube was discarded which containing the flow through solution. Then the column was placed into a new 2 ml collection tube.
- Step (5): 500 μl of wash buffer I (with ethanol added) were added and centrifuged for 1 min at 10,000 rpm then the flow was discarded through and the column was placed back into the collection tube.
- Step (6): 500 μl of wash buffer II (with ethanol added) were added to the column and Centrifuged for 3 min at 15,000 rpm.
  - The collection tube was discarded that containing the flow-through solution and the column transferred to a sterile 1.5 ml micro centrifuge tube.
- Step (7): 200 μl of elution buffer were added to the center of the column membrane to elute genomic DNA and were incubated for 2 min at room temperature and centrifuged for 1 min at 10,000 rpm.
- Step (8): The purification column was discarded and the purified DNA was stored at -20°C. (Thermo Fisher Scientific Inc., 2016)

**Reagents:**

- PCR Master Mix: Cosmo PCR Red M.Mix (2x) (LOT no. o4fp71179) which allow PCR products to be loaded directly into a gel without need to add loading buffer (Bioline Reagents Ltd., USA)
- Tris-Borate EDTA buffer stock (10xTBE): (Biomatik, Canada).
- Ethidium bromide (10 mg/ml): (Biomatik, Canada).
- 2% Agarose gel: 2 gm agarose (Biomatik, Canada) were added to 100 ml 1x TBE buffer in a conical flask and dissolved by heating on magnetic starring. The solution was allowed to cool to around 50ºC, then, 5 μl of ethidium bromide solution (10 mg/ml stock) were added. The mixture was mixed by swirling, poured into a gel-moulding tray with a pre-placed comb and allowed to set till the gel became solid (about 1/2 hour).
- Deionized water (RNase/DNase free): (Biomatik, Canada).
- Glycerol loading buffer: (Biomatik, Canada).
- DNA marker: 100 bp ladder (Biomatik, Canada)

**PCR conditions:**

The following PCR mixture was prepared.

- MyTaq™ Red Mix (2x) 12.5μl
- Forward Primer (20 umol) 1 μl
- Reverse Primer (20 umol) 1 μl
- Deionised water (RNase/DNase free) 7.5μl
- DNA 3μl
- Total volume 25μl

**Cycling conditions:** were different

- **Multiplex reaction mixes**: The temperature profile started with five minutes at 94 ºC followed by six cycles at 94 ºC for 30 s, 67 ºC for 40 s (touch down for 0.5 ºC per cycle) and 72 ºC for 50 s. Subsequently, 27 cycles at 94ºC for 30 s, 64 ºC for 40 s and 72 ºC for 50 s were added. The protocol ended with a final step at 72⁰C for two minutes**.**
- **D antigen**: 95⁰C for 5 min; 35 cycles of 1 min at 95⁰C, 1.5 min at

49⁰C and 2.5 min at 72⁰C; and finally 1 cycle of 9 min at 72⁰C **.**

- **(C, c, E, and e) antigens**: 94ºC for 10 min, 30 cycles at 94ºC for 30 sec, 58ºC for 45 sec and 72ºC for 45 sec,5 min at 72º.
- The amplification reaction was carried out in Veriti ^TM^ 96 well thermal cycler (Applied Biosystems, Foster city, CA).
